# The Principles and Practice of Surgery

**Published:** 1852-10

**Authors:** 


### REVIEWS.

---

ART. III.—*The Principles and Practice of Surgery.* Illustrated by three hundred Engravings on Wood. By WILLIAM PIRRIE, F. R. S. E., Regius Professor of Surgery in the Marischal College and University of Aberdeen; Surgeon to the Royal Infirmary, &c., &c.

We referred very briefly to this work in our last number, and gave the impression that a superficial examination of it had made upon our minds. A more extended and careful inspection of its varied contents has confirmed that favorable impression, and we have no doubt that it is destined to become, in a very short time, one of the most popular text-books on Surgery extant. This American reprint of the English edition has been improved by the editor, Dr. Neill, who has not only increased the number of illustrations, but added several new articles to the original of peculiar interest to the American student. The subjects treated of in the volume are, inflammation and its results; erythema and erysipelas; wounds; burns; fractures; injuries of the head; dislocations; affections of the osseous system; diseases of joints; curvatures of the spine; talipes; affections of the arteries and veins; hernia; wounds of the abdomen; calculous disorders; affections of the testicle; affections of genito-urinary organs; amputations and resections; deligation of arteries; affections of rectum; affections of the eye and its appendages; affections of nose; affections of mouth, throat and windpipe; tumors; and affections of breast; to each of which a chapter is devoted, twenty-five in all. The illustrations are exceedingly well given, and impart a higher value to the work. In a word, it strikes us as being precisely the work wanted by students on this subject. We proceed to notice a few points discussed by the author at such length as our limits will permit.

In reference to the origin of erysipelas he remarks, that while it comes on in many instances without any obvious cause, it often spreads by means of contagion—a remark, the truth of which, though the French authorities generally deny it, will be questioned by few American physicians of experience. It is curious to observe, indeed, how the doctrine of the transmission of disease by this agency is gaining ground in our country. A few years ago, the popular doctrine of our schools was, that only small-pox, syphilis, and a few other complaints, were unequivocally contagious. The communicability of measles, scarlatina, erysipelas, &c., by this method, if not openly denied, was strongly questioned by our highest authorities. But at the present time few teachers are to be found who entertain doubts on this point; and cholera, typhus and typhoid fever, seem to be passing rapidly into the same category.

Mr. Pirrie speaks of a "periodic erysipelas," of which, we presume, the experience of most of our senior brethren has furnished them with examples. He says:

The peculiarity of this form is not merely that it returns, but that it is sometimes strictly periodical, returning more frequently (so far as my observation has enabled me to form an opinion) to the parts which were previously attacked; in some instances it has been found to be periodical in return, and universal in extent—that is, extending over the whole body; an example of which occurred in the experience of Mr. Maul, of Southampton, in the case of a lady who had several attacks at intervals of two years. In some instances the attack is monthly, at the time the catamenia should appear. This form of erysipelas is, I believe, most frequently met with in females of a weak and chlorotic habit. I have a patient who for some years had a return of it every six weeks, but got over the tendency by a residence in the country, and the use of suitable remedies for the general improvement of her health and strength. I know an instance of a man who had for years an attack every two months. In both the last-mentioned cases, the head was the part attacked. One man, whose case I remember, had an attack regularly twice a year, and another every spring. The local symptoms, so far as I have had an opportunity of observing, are not very severe, and rarely give rise to more than œdema of the cellular tissue, and exfoliation of the cuticle. The constitutional symptoms are both antecedent and attendant.

The chapter on *wounds* is full and satisfactory, presenting in small compass a summary of what the practitioner is desirous to know. Mr. Pirrie does not limit himself to mere surgical matters, but touches upon points of physiology and pathology relating to his subject, and thus gives to his work a philosophical character which renders it more eminently worthy of general acceptance. In this chapter he discusses the reparative process in the following terms:

*The permanent tissues of repair.*—With regard to the repair of injuries, it is known that in healing, the lesion of some textures is effaced by the reproduction of similar tissue, while the injury of other parts is repaired by the formation of a tissue less highly organized. Osseous and cellular tissue may be reproduced, and minute nervous and vascular filaments are formed in the connecting substance. The development of blood-vessels for granulations, or for superficial deposits of lymph, adhesions, or the like, has been referred to in the chapter on Inflammation, and the views entertained by many regarding their formation, have been mentioned; but in addition to the opinions already brought forward, it is proper to state that, according to Mr. Paget, their development is always effected by the projection of *culs de sac*, commencing as mere dilatations, from a capillary arch passing close to the adventitious structure. These cœcal diverticula, crowded with corpuscles, are prolonged in a definite manner, towards and into the new tissue, so that they meet and adhere ; the double partition formed by apposition of their closed extremities gives way, and a new capillary arch, transmitting blood, is formed. Fibrous tissue is the medium of repair in wounds of cartilage, in the cut extremities of which, however, bone is sometimes deposited. Muscular fibre, when divided, is never reproduced, cellular and fibrous tissues forming the new bond of connexion, which gradually contracting, in most instances, draw the retracted ends of the muscles at last into pretty close apposition. Nerves, when divided, if their cut extremities be in contact, rapidly unite, but with some confusion of function, apparently from the precise continuity of individual fibres not being accurately restored. Even when a considerable interval has occurred between the ends, union has been effected, in the first place by material similar to that effused after wounds of other soft tissues ; but, in time, nerve-fibres become developed within this substance, probably by prolongation from the cut extremities, between which they form a communication, partially restoring the functions of the nerve. Not less, it is believed, than two years will suffice for the accomplishment of this process. There is "no example in which the nerve or ganglia corpuscles have been reproduced." The repair of wounds differs somewhat according to their amount of exposure.

The subject of fractures is very fully illustrated. The surgeon will find a representation of all forms of injury likely to be met with, and of all the appliances required in this branch of his art. The same may be said of the chapter on injuries of the cranium. Our author gives the following as the *conditions which justify the operation of trephining :*

From what has been stated regarding injuries of the head, it will appear that operative interference is thought to be justifiable under the following circumstances:—

1. In simple fracture with depression, provided the symptoms persist after the use of depletion, purging, and other alleviating remedies.

2. In compound fracture with depression, without symptoms of compression.

3. In punctured fracture, without symptoms of compression.

4. When the symptoms are very urgent, and the surgeon thinks he has good reason to believe, that they are caused by blood or purulent matter underneath the cranium and above the dura mater, or by fracture with depression of the inner table.

In each of the first three conditions, it is considered necessary to adopt proceedings for raising the depressed portion of bone to its proper level. If, on exposing the fracture, it is found that this cannot be done by means of the elevator, which is often the case, in consequence of the fractured portions being so related that it is impossible to insinuate the extremity of the elevator underneath the depressed portion, then it is advisable to remove a small portion of the cranium by means of the trephine, for the purpose of allowing the introduction of the elevator by which the depressed part is to be raised. The instrument used by the ancients was the trepan, and the operation was called trepanning; the instrument now used is called the trephine, and the operation, trephining.

Such are the views now entertained in these latter and better times of surgery, as to the conditions which justify and require the operation of trephining. We find, however, from the history of surgery, that a very different doctrine prevailed in former days; that so great was the rage for trepanning among the ancients, that the very slightest fissure, or even the mere suspicion of one, was considered to be sufficient warrant for the operation; that they trepanned in all fractures, whether attended by depression or not, whether attended by symptoms of compression or otherwise; that they operated when bad symptoms were present, to remove them—when absent, to prevent them; so that they elevated every depression, trepanned every fracture, and, in operating on a longitudinal or a radiated fracture, they trepanned along the whole of its course, so as to saw it out; and did not allow a single fissure of it, or rima, as they called it, to escape- In operating for the removal of a coagulum, they made as many openings as would uncover, if possible, the whole of it ; and, says Ravaton, "I have seen surgeons so infatuated, so desperately bent on discovering abscess of the dura mater, that, after applying six crowns of the trepan, they would, and I verily believe have, pulled away all the remaining bones of the cranium, had not their patients been delivered by death from such operations." All this was done, they said, to remove danger. It is almost incredible to what a disgraceful extent this fashion for trepanning was allowed to outrage common sense ; and it is difficult to imagine how they could believe a fracture to be so dangerous, and their operations so safe. To show the extent to which trepanning was carried, Mr. John Bell gives the following quotation :—"Godifredus, Chief Surgeon of the States of Holland, mentions with particular exultation the performance of this operation by his friend, who trepanned the cranium of the Count of Nassau twenty-seven times, and that the fact might be established on indisputable authority, he made the said Count of Nassau, after he was recovered, write the following curious certificate, on the 12th day of August, 1664: 'I, the underwritten, Philip Count Nassau, hereby declare and testify that Mr. Henry Chadborn did trepan me in the skull twenty-seven times, and after that did cure me well and soundly.'" These practices, and the numerous inventions of instruments for cutting the skull, are sad monuments of the surgery of past ages. In later times, the Royal Academy of Surgery in France revived and defended the doctrine that all fractures of the cranium ought to be trephined. It does seem surprising that this body of men, convened for the purpose of ascertaining the principles which ought to regulate the practice of our science, and to whom surgery is in other respects so greatly indebted, shonld, by giving the sanction of their high authority to so dangerous a doctrine, have led the young members of our profession to adopt very dangerous rules of practice. The unfortunate results of the operation were so numerous, that the celebrated Desault, one of the greatest ornaments of surgery in France, forming his opinion from what he saw at the Hôtel Dieu, strongly condemned the practice, and, in the latter part of his life, entirely discarded it. The doctrine of the French Academy met with a most powerful advocate in this country, in the late Mr. Pott, who, with all the great talent and decision for which he was so eminently distinguished, maintained the doctrine of trephining in almost every fracture, to prevent as well as to remove bad symptoms. He states that fracture of the skull in many cases is not attended with any symptoms actually demanding this operation at the moment; but although there may be no symptoms denoting affection of the membranes, or of the brain itself, yet inflammation in those parts will, in consequence of the fracture, come on at a more or less remote period, and that, therefore, recourse ought to be had to the operation. Mr. Pott, in speaking of the doctrine of trephining, says, "I am as much convinced of this as of any fact which repeated experience may have taught me," and throughout his writings on that subject, he gives his opinion with so much decision, supporting it by cases and arguments, that his views cannot fail to have produced a very decided impression. Notwithstanding, however, all the eloquence and talent with which he supported his views, the doctrine he taught is now abandoned; and in these later and milder times of surgery, operative procedure is considered justifiable only under the circumstonces mentioned at the commencement of this section.

This extract will recall to the mind of the reader some excellent remarks on this subject by Dr. Hardin, in the March number of this Journal, in which the ground was taken that trephining should be performed in cases of depression, whether symptoms of compression were present or not. We entirely concur in the views of Dr. Hardin, and while we should deprecate that reckless resort to the instrument which so disgraced early surgery, we hold that the timidity is to be avoided, which, to shun a slight danger, subjects the patient to the peril of epilepsy, mania, and fatuity. Depression cannot be continued without imminent risk of these frightful consequences.

On the subject of dislocations, Prof. Pirrie treats fully, clearly, and ably. Referring to the auxilliaries in the reduction of dislocated joints, he says, "Chloroform is the most powerful, and it is now invariably preferred. It prevents pain, facilitates reduction, renders comparatively little force necessary, thereby diminishing the danger of injuring texture, and it leaves no permanent weakness of system."

In his chapter on "affections of the osseous system," he gives an account of periostitis, the idiopathic form of which was first clearly described by Sir Philip Crampton. The exciting causes of this form of the disease, are, "atmospherical influence and mechanical injury; the predisposing causes, a feeble, debilitated state of body, induced by mental anxiety, or long-continued derangement of the digestive apparatus." As to treatment, he remarks:

In the acute form, the constitutional treatment consists of low diet, general depletion, saline purgatives, diaphoretic medicines, and such means as are capable of procuring resolution. The local treatment includes quiet, an attitude favorable to the reflux of the venous blood, leeches, warm and emollient applications, as fomentations, poultices, and other antiphlogistic means. Free division of the periosteum should be employed, according to some, only when other treatment has failed. Professor Miller objects to free direct division if suppuration be not present, and recommends a valvular division of the inflamed periosteum. Professor Syme says, "The mode of treatment depends upon the intensity of the symptoms. When they are very violent and attended with smart fever, the most effectual practice is to make a free incision through the inflamed parts down to the bone. When less severe, no benefit is derived from this proceeding."

When chronic, it is to be treated by internal alterative remedies, as hydriodate of potass, of which ten grains should be given during the day; or, when this fails, the bichloride of mercury, for, singular as it may seem that, this mineral, which is a predisposing cause of periostitis, should prove a remedy, it is, nevertheless, "an ascertained fact." Upon this point Mr. Lawrence is quoted by our author to the following effect:

I have seen, in so many instances, the pain in that disease continue unrelieved, in spite of the pretty active employment of local antiphlogistic means, in spite of mild mercurial treatment, and have so constantly found it yield only to the full influence of mercury on the system, that I own myself to be at a considerable loss to the opinion entertained by many, that inflammation of the periosteum and affections of the bone are actually brought by the use of mercury. It seems to be very inconsistent that one and the same medicine should be capable of decidedly relieving inflammation of a certain texture. I think I formerly had occasion to mention that I did not coincide with the opinion of many, that those states were produced by the mercury, and certainly, when speaking of inflammation of the periosteum, whether arising from syphilis or not, I do not know of any means so capable of relieving the disorder as mercury.

The joints are subject to many singular departures from a healthy condition, and illustrations of strange morbid action are given in the chapter which treats of these affections. It is a highly instructive chapter. The editor adds a section on 'hysterical affections of the joints," which we copy:

Hysterical females often complain of great pain in the joints, which might be mistaken for some real and dangerous disease of the part. According to Brodie, "At first there is pain referred to the hip, knee, or some other joint, without any evident tumefaction; the pain soon becomes very severe, and by degrees a puffy swelling takes place, in consequence of some degree of serous effusion into the cells of the cellular texture. The swelling is diffused, and in most instances trifling; but it varies in degree; and I have known, where the pain has been referred to the hip, the whole of the limb to be visibly enlarged from the crista of the ilium to the knee. There is always exceeding tenderness, connected with which, however we may observe this remarkable circumstance, that gently touching the integuments in such a way as that the pressure cannot affect the deep-seated parts, will often be productive of much more pain than the handling of the limb in a more rude and careless manner. In one instance where there was this nervous affection of the knee, immediately below the joint, there was an actual loss of the natural sensibility; the numbness occupying the space of two or three inches in the middle of the leg. Persons who labor under this disease are generally liable to other complaints, and in all cases the symptoms appear to be aggravated, and kept up by being made the subject of constant anxiety and attention."

*Treatment.*—Unfortunately for the patient, this affection is sometimes treated for a diseased or injured joint by antiphlogistic measures, which necessarily aggravate the symptoms. Constitutional remedies are those to be relied on. All of the functions are to be restored to a healthy condition, particularly the menstrual and digestive, since these will be usually found at fault.

Hygienic remedies are more valuable than local. "The patient should have fresh air, generous living, and plenty of occupation for mind and body; she should be encouraged to take exercise, notwithstanding pain and weakness; and to resume, as far as possible, the habits of a healthy person." The shower-bath and frictions of the skin will improve the capillary circulation. Tonics, such as quinine, valerian, and iron, will be found most valuable where there is debility. Brodie has also found benefit in the use of assafœtida injections, and in the enveloping the joint with a plaster composed of equal parts of the extract of belladonna and soap plaster.

Curvatures of the spine forms the subject of the next chapter, and in this connection some interesting physiological remarks occur:

It may be regarded as a general law, that of two functions, voluntary motion and sensation, the former is almost invariably first removed, and the latter first restored; the rationale of which is, that the anterior columns of the spinal cord, which give off the anterior roots ot the nerves, by which they preside over voluntary motion, are nearer to the seat of the disease, and therefore more exposed to pressure than the posterior columns which give off the roots presiding over sensation. Although pressure on the spinal cord is usual in angular curvature, it is surprising how nature, even in some cases where the destruction is very great, yet continues to maintain the integrity of the vertebral canal, so as to prevent the cord from being compressed. Of many examples of this remarkable fact, I shall only refer to the following: Mr. Stafford mentions the case of a child, in whom, though the bodies of six dorsal vertebræ were destroyed, and the angle of the curve was very acute, paralysis did not occur. Professor Cruveilhier gives the particulars of a case in which the bodies of five dorsal vertebræ were completely destroyed; where the fifth dorsal vertebræ rested on the eleventh, the two becoming anchylosed, and at a very acute angle; and yet the medulla was preserved free from pressure. I have at present under my care a girl ten years of age, in whose case the bodies of the fourth, fifth, sixth, and seventh dorsal vertebræ must be entirely removed; an abscess is formed, and is pointing about the middle of the seventh rib; and judging from the appearance of the spine behind, the parts above and below the seat of the disease must be for a short distance almost parallel with one another, so abrupt is the curve; and still the patient is as yet quite free from any symptoms of compression of the spinal cord. The only explanation given of such cases is that the process of destruction must have been very slow, and the deviation from the natural form extremely gradual. Mr. Stafford remarks: "The completeness and incompleteness also of the symptoms very much depend upon the rapidity with which the curve takes place. If the destruction of the bodies of the vertebræ has been very quickly effected, the paraplegia is usually more complete; but, if it has been slow in its progress, the paralysis below is often very imperfect."

On the treatment of these deformities he has the following remarks:

Any attempt to remove the curvature would be most injudicious. Anchylosis is the only favorable termination to be hoped for, and therefore the object aimed at in treatment should be, to place the patient under circumstances most likely to conduce to that result. With that view, it is indispensable, first, to keep the patient in a recumbent position, so as to remove from the diseased parts the pressure of the superimposed weight, and to preserve the parts as much as possible in a state of perfect quietude in that position; and secondly, to use all means, judicious and available in the circumstances of the case, for maintaining the general health. In some cases local remedies are highly beneficial.

That it is necessary to confine the patient to the recumbent position, does not admit of question, for it is evident that the superimposed weight pressing on the diseased part not only acts as a source of irritation, but must tend to increase the curvature; and it can only be effectually removed by placing the body in the horizontal position. And that any effort which nature may make to effect anchylosis may not be defeated, it is further necessary that the parts should, as much as possible, be prevented from being moved upon each other. Another advantage which results from preserving the parts at perfect rest in the horizontal position, is, that the removal of the irritation, caused by the superincumbent weight, from the diseased parts, diminishes the danger of the formation of abscess, which (as formerly stated) is a most unpromising occurrence, and must induce the gloomiest apprehensions as to the ultimate results. One of the best means for fulfilling the above indications, is, to place the patient in the supine position on Earle's bed, which, besides other advantages, rendering it very convenient for this part of the treatment, allows the relative position of the trunk and limbs with regard to each other to be slightly changed, without any risk of moving the diseased parts on each other. The slight change thus allowed, renders the confinement to the recumbent position much less irksome than otherwise it would be. As an additional precaution from preserving the diseased parts from any movement, it is, in many instances, advisable to apply splints on each side of the spine. The splints, in such eases, must suit the shape of the parts to which they are applied. Some recommend the patient to be placed in the supine posture, but others give the preference to the prone position, because in that attitude the superimposed weight is more effectually removed—there is no risk of heat and irritation from pressure—it favors the return of venous blood from the bodies of the vertebræ—and the approach of paralysis, it is thought, may be deferred, as matter will gravitate away from the medulla. This position is also very convenient when local applications are necessary, and in some cases the curve is so abrupt, that it is almost impossible, with every precaution, to keep the patient long on his back without producing irritation of the soft parts. But notwithstanding the above-mentioned advantages, I confess I have, in the majority of cases, found treatment conducted in the supine posture more satisfactory, and chiefly, I believe, from the diseased parts being more easily preserved in a state approaching to complete immunity from motion, than is possible when the treatment is conducted with the patient in the prone position, in which I have often been annoyed by finding it impossible to prevent the patient from moving the upper part of the spine by frequently moving the head and shoulders; and, as far as my experience goes, the supine position is preferred by patients. Rest, however, of the diseased parts, and the recumbent position, whether the body be prone or supine, are of the utmost importance from the very commencement of the disease, until a cure is effected by anchylosis. When it is believed that anchylosis has taken place, and the patient is allowed to resume the erect attitude, it is a judicious precaution to employ for some time an apparatus, such as that generally known by the name of the spine supporter, for removing the superincumbent weight.

The maintenance of the general health is another and equally important indication, but unfortunately some of the best means for fulfilling it are not compatible with the rest and the recumbent position which form essential parts of judicious treatment. The great importance of attending to the general health must be evident, when it is considered under what circumstances scrofulous deposits are most apt to take place in bone. In individuals of a scrofulous diathesis, insufficient nutriment or clothing, living in damp or cold and impure atmosphere, want of exposure to the sun's rays, mental depression, and any cause of debility acting permanently or habitually for a length of time, have unquestionably an influence in exciting scrofulous deposits in bone, as well as in other textures. These considerations suggest the necessity, especially in scrofulous cases, of a digestible diet, living in a pure dry atmosphere, (the bracing air of the seaside being often highly beneficial,) exposure to the light of the sun, the cultivation of habitual cheerfulness, the proper regulation of the digestive apparatus, and the use of such remedies as from the particular circumstances of the case are best calculated to improve the general health. The tonic medicines generally found most useful are the preparations of iron. But as far as medicine is concerned, I believe the most important point is, to have recourse to those remedies which, from the particular circumstances of the case, seem most likely to preserve the digestive organs in a proper state. Besides these means, in some cases, local remedies are necessary; but the employment of them will depend upon the nature of the cause of the disease. If the disease depend upon scrofulous caries of the vertebræ, or upon softening with absorption without ulceration or caries, depletion would be worse than useless, and would tend to weaken the patient. In these cases, the surgeon must content himself with advising the recumbent position, maintaining the diseased parts in a state of quietude, and prescribing all suitable means for preserving the general health. In scrofulous caries, benefit will often be found to accrue from the early and very cautious employment of counter irritation, along with the treatment here alluded to. If the curve arise from inflammation of the bodies of the vertebræ, of their investing membrane, or of the intervertebral cartilages, slight local depletion by leeching or cupping at the commencement of the disease, and afterwards counter-irritation, are known to be highly beneficial. The repeated application of small pieces of blister to each side of the vertebral column at the seat of the disease has been found well suited for children, and caustic issues for adults.

A chapter is devoted by Mr. Pirrie to Talipes, the cure for which is one of the triumphs of modern surgery; for from the time of Hippocrates until a period within the memory of surgeons now living, the treatment of those deformities consisted in the employment of mechanical pressure. The following history of the discovery is interesting:

In 1784 the first step was taken towards the present method, a physician of the name of Thilenius having suggested the division of the tendons affected with shortening; and his suggestion was carried into practice by Lorenz, a surgeon at Frankfort, in the case of a young lady affected from infancy with Talipes varus. The heel descended two inches after the operation, and the lady was able to walk on the entire sole. It was the tendo Achillis that was divided, and the operation was performed under the direction of Thilenius. The division, however, was effected by a large wound. The suggestion of Thilenius was also carried into practice at a subsequent period by Sartorius, whose method of operating, however, was liable to many objections, particularly because he made extensive incisions of the superimposed parts, exposed the tendon at the part to be divided, and after section of the tendon, immediately attempted to bring the foot to its proper position. The consequences were unfavorable, and such as do not follow the operation of section of the tendons as it is practised at the present day. Michälis suggested a different method of treatment, which consisted in a partial division of the shortened tendon, and bring the foot to its proper position immediately after the operation; he recommended section of the tendon to the extent of one-third of its thickness, by which means its strength would be materially diminished. He performed his first operation in November, 1809. Both Sartorius and Michälis recommended immediate restoration of the foot to its proper position, the one after complete, the other after partial division of the tendon; but neither of their methods of treatment met with general adoption or approval.

Delpech had the merit of conceiving and recommending some new and important principles of treatment. Having observed that after rupture of the tendo Achillis and some other injuries, the uniting medium admits of considerable elongation, it occurred to him that the same elongation could be obtained after section of the tendo Achillis for the cure of Talipes, provided mechanical extension be employed before the uniting medium has acquired great strength and firmness. He recommended that the section of the tendon should be effected without division of the common integument over it; and in the instance in which he performed the operation, he made a wound of the common integument an inch in length on each side of the tendo Achillis by passing a scalpel between it and the deeper-seated structures; and then by a convex edged bistoury, he divided the tendon from before backwards. He recommended that after the operation the cut portions should be preserved in apposition, by maintaining the foot in the distorted position by mechanism, until reunion of the divided tendon be effected—that careful and gradual extension of the uniting medium should then be made, until the tendon be of sufficient length; and this having been obtained, that the limb be kept in a proper position by means of an apparatus until the new substance has acquired sufficient strength. Delpech performed his operation in May, 1816, and although a cure was effected after a long period, and the patient ultimately recovered, yet there were so many discouraging circumstances, that he never repeated his operation. Although the mode of dividing the tendon recommended by Delpech is exceedingly objectionable, he certainly has the merit of having suggested some important principles in the after treatment. To Stromeyer, who performed his first operation in February, 1831, the praise belongs of having perceived what was objectional, and appreciated what was valuable, in the views of those who preceded, and of proposing and performing the safe and successful operation, and the mode of after treatment, which are now so generally approved of, and so successfully adopted.

The chapter on calculous disorders has been more enlarged by the editor than any other in the volume. He has copied freely from the late work of Prof. Gross on "Diseases and Injuries of the Bladder." It will be found to comprise whatever is most practical and important in relation to those interesting subjects. Western America undoubtedly abounds more than any other quarter of the globe in calculous disorders. It is to our surgeons and physicians that the profession will look for the statistics which are to elucidate the pathology of these affections. It is in our region that the most remarkable feats have been performed in the extraction of urinary calculi. Western surgeons have unquestionably operated for stone in the bladder with greater successs than any others in the world.

In the chapter on affections of the eye and its appendages, Prof. Pirrie makes some interesting remarks in reference to strabismus, about which there was so much said in the journals a few years ago, and the operation for which, from having been so popular, seems suddenly to have fallen almost into disuse. We can see no reason for this sudden loss of popularity. No doubt the operation was sometimes unsuccessful. There are cases in which it ought never to be performed. Prof. Pirrie says:

Cases of strabismus proceeding from gastric, intestinal, or uterine derangement, from general debility, or [from affections of the brain, are all unfit for operation; as, also, are all cases in which the deformity is in consequence of opacity in the cornea, the distortion in such instances being an effort to remove the opacity from the axis of vision.

But in proper cases, as those "in which the strabismus is confirmed, and in which there is no evidence of an exciting cause calculated to keep up or bring back a disturbance in the equilibrium of muscular action within the orbit," there is no reason why it should not succeed. In all such cases, when correctly executed, it does succeed, and there are few operations which are attended with more gratifying results than that for strabismus.

Imperfect as this notice is, it must suffice for this admirable treatise. We could easily have filled our journal with extrrets from its pages which our readers would peruse with interest ; but the space at our command is exhausted, and we dismiss it with the assurance that they cannot purchase a more valuable work on surgery.

